# Material needs security and mental health outcomes in adults with type 2 Diabetes in Lebanon: A cross-sectional study

**DOI:** 10.1007/s40200-024-01484-6

**Published:** 2024-08-16

**Authors:** Ola Sukkarieh, Maya Bassil, Leonard E. Egede

**Affiliations:** 1 Rafic Hariri School of Nursing, American University of Beirut, Beirut, Lebanon; 2 Human Nutrition Department, College of Health Sciences, QU Health, Qatar University, P.O. Box 2713, Doha, Qatar; 3 Department of Medicine, University at Buffalo, Jacobs School of Medicine and Biomedical Sciences, Buffalo, NY USA

**Keywords:** Type 2 diabetes mellitus, Social determinants of health, Diabetes fatalism, Depression, MENA region

## Abstract

**Objectives:**

Despite their documented significance in type 2 diabetes (T2DM) management, social determinants of health (SDOHs) including material needs security and mental health outcomes are understudied in the Middle East and North Africa (MENA) region. This study aims to assess the relation between material needs security and mental health outcomes in Lebanese adults with T2DM.

**Methods:**

Subjects with T2DM (*N* = 300) were recruited from primary health care centers in Lebanon. Sociodemographic, material need variables, depression measured by Patient Health Questionnaire (PHQ-9), and diabetes fatalism measured by Diabetes Fatalism Scale (DFS) were collected.

**Results:**

Most of the participants were men, married and with lower educational levels. Multivariate analyses revealed that having material needs security was associated with diabetes fatalism (β = -0.63(-1.13; -0.12)), and depression (β = -0.46(-0.78; -0.13)). Also, higher age (β = 3.49(0.91; 6.06)) and education (β = 3.42(1.18; 5.66)), and lower income (β = 3.25(0.62; 5.88)) were independently associated with diabetes fatalism. Being male was the only independent variable associated with less depression (β = -1.56(-2.97; -0.14)).

**Conclusion:**

Our study highlights the importance of material needs security on mental health outcomes for adults with T2DM in the MENA region. Clinicians are encouraged to assess the impact of material needs on mental health outcomes. Further research is needed to understand potential pathways/mechanisms and options for effective interventions and policymaking.

## Introduction

Type 2 diabetes mellitus (T2DM) is a fast-growing pandemic with substantial health complications. The prevalence of T2DM is affecting low- and middle-income countries more widely compared to affluent industrialized countries [1]. Emerging research indicates an emphasis in understanding population health outcomes where social determinants of health (SDOH) has surfaced as an integral framework to improve overall health [2, 3]. The World Health Organization (WHO) defines SDOH are circumstances of living, learning, working, and leisure conditions that may affect patients’ health risks and outcomes. In 2013, the American Diabetes Association (ADA) endorsed, in a scientific statement, the need to understand SDOH to curb T2DM epidemics and delay or prevent diabetes related complications [4]. Understanding SDOH such as formal education, health literacy, neighborhood safety and cohesion, behavior and social support, and the ability to meet material needs in T2DM plan of care are significantly associated with sustained clinical outcomes, self-management and access of care [4, 5].

Material needs, a subset of SDOHs, encompass the fundamental needs for maintaining health and managing illness. Examples include telecommunication, transportation, housing, utilities, food, medication, and confidence in filling health forms [6]. Material needs insecurities, on the other hand, indicates lack of sufficient resources to maintain health and mitigate illnesses for individuals [6]. In diabetes context, material needs insecurities have been associated with poor care utilization [7], high out of pocket expenses despite having health insurance [8] as well medication underuse and lack of food which ultimately led to poor diabetes outcomes [6–9]. Psychological aspects of SDOHs explore the interaction between individuals and diabetes outcomes [10]. Empirical data has shown the strong interrelationship between depression and adverse diabetes outcomes and self-care behaviors [10, 11]. Currently, the literature is unfolding a strong co-existence between poor housing conditions and depression and the adverse diabetes outcomes [10–12]. An additional construct that has affected diabetes outcomes is diabetes fatalism, which is defined as “a complex psychological cycle characterized by perceptions of despair, hopelessness, and powerlessness” [13]. Increased diabetes fatalism was associated with decreased medication adherence and self-care behaviors and poor diabetes outcomes [13, 14]. A plausible explanation to such relationships is that when individuals live in dire circumstances such as poor housing conditions and lack of material securities and access to proper care, fatalism rises to compensate for any form of loss [12].

Lebanon is a country in the MENA region with one of the highest diabetes prevalence, with current estimates standing at around 9% [15]. In addition, Lebanon is one of the low middle-income countries in the MENA region that has been undergoing detrimental socioeconomic turmoil impacting all non/governmental sectors profoundly. As a result, material needs insecurity may be on the rise, and so is the decline in mental health outcomes. Additionally, determinants rooted in the cultural background of individuals, such as fatalistic beliefs, and psychological status towards disease have also been correlated with disease outcomes [2, 13, 14]. Though a variety of socioeconomic and psychological factors have independently been shown to influence diabetes outcomes, the incremental influence of each of these factors on diabetes outcomes in the Lebanese adult population is unknown. We herein refer to depression and diabetes fatalism collectively as mental health outcomes. Therefore, the aim of this study was to collect primary data on social determinants of health and examine the independent association between material needs insecurity and mental health outcomes for T2DM.

## Methods

### Design and participants

A cross-sectional study with a sample of 300 Lebanese participants with T2DM recruited, using convenience sampling, over a period of three months in 2019 from three primary health care centers (PHCs) in Lebanon (Beirut, Mount Lebanon, and North Lebanon) targeted to have diverse sociodemographic characteristics of participants with T2DM. Participants were recruited from patients that were present at the PHCs at the time of data collection, or were scheduled through appointments made by phone calls, and those interested were provided an explanation of the study prior to being consented. Eligibility included age 18 years or older, confirmed in the medical charts with diagnosis of T2DM, and ability to communicate in Arabic. Subjects were ineligible if they had documented psychiatric diagnoses or showed mental confusion during the interview, or they had reported alcohol or drug abuse/dependency, dementia, active psychosis, or acute mental disorders. After providing written consent, data was collected and included questionnaires. Clinical judgment was employed to assess the participants’ capacity to comprehend the consent form and questionnaires. This assessment included back-and-forth discussions to ensure understanding and provide further validation. Specific criteria were used to evaluate comprehension, such as the participants' ability to explain the content in their own words and respond accurately to related questions. Minimal number of subjects were excluded accounting for less than 5% of the overall sample size. After providing written consent, participants were asked to fill out study questionnaires.

#### Sample size

Sample size calculation for structural equation modeling (SEM) is based on 10:1 ratio for calculation of sample size is suggested, i.e. a minimum of 10 participants for every covariate. Therefore, consistent with the study of Walker and colleagues [5] and having around 20 covariates; the total sample required is 200. After accounting for potential non-response, missing data and drop-out rate, the sample size was inflated by 1.5; equivalent to a total sample of 300 participants.

### Data collection

#### Demographics

Participants completed demographic characteristics through a self-reported questionnaire including years, sex, marital status, education level, employment status, monthly household income in USD, availability of health insurance, and confidence in filling medical forms. Age was computed both as both a continuous and categorical variable (18 – 49, 50 – 64, 65 – 94 years), income was computed as categorical (< 500$; 500$—1499$; ≥ 1500$; not reported), while the other variables were computed as dichotomous (yes/no).

#### Material needs security

A self-report questionnaire was used to assess material needs security variables. Questionnaire included: owning a home, having electricity, television, cable, telephone, air conditioner, heater, wireless-internet, computer, refrigerator, and owning a car as means of transportation. All material needs security questions had a “yes” and “no” response. The variables were coded as dichotomous variables with “yes” = 1 and “no” = 0. A summary material needs security variable was created by summing all the items and creating a continuous variable with a minimum score of 0 and a maximum of 11.

##### Diabetes fatalism

Diabetes fatalism was assessed using Diabetes Fatalism Scale (DFS)-Arabic, defined as ‘a complex psychological cycle characterized by perceptions of despair, hopelessness, and powerlessness’ and associated with poor glycemic control [16]. It is a 12-items questionnaire (DFS12) with three subscales; emotional distress, perceived self-efficacy, and spiritual coping [17]. Higher scales indicate higher fatalism that has been previously validated in the Lebanese population. The DFS-Arabic has a Cronbach’s alpha of 0.77 [16] which is comparable to the original version’s Cronbach’s alpha of 0.80 [17].

##### Depression

Depression was assessed using Arabic PHQ-9, a 9-item scale based on the DSM-IV criteria for depression with higher scores indicating higher depression [18]. PHQ9 scores range between: 0–4 non to minimal, 5–9 mild, 10–14 moderate, 15–19 moderately severe, 20–27 severe. Arabic PHQ-9 was used validated previously with same Lebanese population and was highly consistent based on reliability analyses (Cronbach’s alpha = 0.88) [19].

### Statistical analysis

Study outcomes are mental health outcomes (DFS and PHQ-9), while exposures and potential predictors are the material needs and their sum. Potential confounders or covariates are age, sex, education, income, marital status, insurance, employment status, and Confidence in Filling out forms. Continuous variables (age, sum of material needs security, DFS and PHQ-9) were expressed as mean ± SD, while categorical variables (age, gender, marital status, education, employment, income health insurance, confidence in filling medical forms, material needs security and PHQ-9 variables) were expressed as counts and percentages. Unadjusted and adjusted linear regression models were run to test for the unadjusted and adjusted associations between sum of material needs security and each of the mental health outcomes (DFS and PHQ-9). For both unadjusted and adjusted regression analyses, outcomes (DFS and PHQ-9) were treated as continuous variables. When PHQ-9 was treated as categorical variable, logistic regression was used. Unadjusted logistic regression examined relationship between material needs security and mental health in Adults with T2DM in Lebanon. In adjusted regression analyses, models adjusted for covariates: DFS or PHQ-9, material needs security, age, sex, education, income, marital status, insurance, employment status, and Confidence in Filling out forms. All analysis was run using Stata v.16. Significance was determined based on a two tailed alpha of *p* < 0.05.

## Results

### Descriptive characteristics of study participants

Table 1 presents the demographic characteristics of study participants. Average age was 60 years (SD = 12.07) and 52% were male. The vast majority (73%) were married, 64% had less than high school education, 55% were unemployed, 53% did not have health insurance and 61% reported a household monthly income below 1500 USD, while 71% reported having no confidence in filling out medical forms. Material Needs Security Variables are also presented in Table 1. Most of the participants (64%) possessed a house, had drinking water (84%) and cable (72%) and internet subscription (60%). Additionally, the vast majority owned most of the appliances such as an air conditioner (61.67%), and a heater (82%), whereas only 37.7% had a computer, a Television (99%), a Telephone (97%) and a refrigerator (93%), and everyone had electricity. However, about half of the participants owned a car for transportation. The average sum of material need security variables was 9 (SD = 2.2) out of 11. The mean DFS score was 35.7 (SD = 7.9), while the mean PHQ-9 score was 7.2 (SD = 5.1), which falls in the “mild depression” category. Notably, only 29% of the sample had scores that classified them as having “major depression”.

**Table 1:** Sample Demographics (*N* = 300)

	M ± SD or %
**Age**	60.3 ± 12.1
**Age (years)**	
18 – 49	17
50 – 64	49.7
65 – 94	33.3
**Sex**	
Female	48
Male	52
**Marital status**	
Married	73
Not married	27
**Education (years)**	
< high school grad	63.8
≥ high school grad	36.2
**Employment**	
Employed	44.7
Unemployed	55.3
**Household Income**	
< 500$	30.3
500$—1499$	30.7
≥ 1500$	10
Not reported	29
**Health Insurance**	
Yes	46.8
No	53.2
**Confident in filling out health forms**	
Yes	28.8
No	71.2
**Sum of Material Needs Security**	9.0 ± 2.2
**Diabetes Fatalism**	35.7 ± 7.9
**Patient Health Questionnaire (PHQ9) Total Score**	7.2 ± 5.1
**Patient Health Questionnaire (PHQ9)**	
No major depression	70.8
Major depression	29.1

^*^Data is presented as mean ± SD for continuous variables and percentage (%) for categorical variables

### Regression models of associations with DFS and PHQ-9 as outcome variables

#### Unadjusted models

The unadjusted relationship between the sum of material needs security and mental health outcomes (DFS and PHQ-9) is presented in Table 2. Higher material needs security was significantly associated with less depression scores (β = -0.61 (-0.86;-0.35)). In addition, when depression was treated as categorical variable, the odds ratio was statistically significant indicating when sum of material needs increases, there is less odds of developing depression 0.81(0.72; 0.91).

**Table 2:** Unadjusted logistic regression of relationship between material needs security and mental health in Adults with T2DM in Lebanon

	Diabetes fatalism* β (95% CI)	PHQ-9 (Continuous)* β (95% CI)	PHQ-9 (categorical)** OR (95% CI)
Sum of Material Needs Security	-0.39(-0.80; 0.01)	**-0.61(-0.86; -0.35)**	**0.81(0.72; 0.91)**

^*^ Linear Regression, Coefficients of associations (95% CI) are presented

^**^ Logistic Regression, Odds ratio (95% CI) is presented

Values in bold indicate statistical significance (*p* < 0.001)

#### Adjusted models

Table 3 shows results from fully adjusted models accounting for relevant covariates. Higher material needs security was associated with less diabetes fatalism (β = -0.63(-1.13;-0.12)), and less depression when treated as continuous variable (β = -0.46(-0.78;-0.13)) and less odds of developing depression when treated as categorical variable (β = 0.82(0.69; 0.95)). With respect to other independent variables associated with higher sum of material needs, being between the age of 50–64 (β = 3.49(0.91; 6.06)), having equal or more than high school education (β = 3.42(1.18; 5.66)), making small income of 500$—1499$ (β = 3.25(0.62; 5.88)) as well as not reporting an income (β = 3.72(1.17; 6.27)) were associated with higher diabetes fatalism. Being male was the only independent variable associated with less depression (β = **-1.56**(-2.97; -0.14)).

**Table 3:** Adjusted regression analyses of the relationship between material needs security and mental health in Adults with T2DM in Lebanon

Variables	Diabetes fatalism* β (95% CI)	PHQ-9 (Continuous)* β (95% CI)	PHQ-9 (categorical)** OR (95% CI)
Age			
50–64	**3.49(0.91; 6.06)**	-0.069(-2.73;0.60)	0.72(0.33;1.56)
65–94	1.13(-1.79;4.05)	-0.90(-2.81;1.01)	0.72(0.29;1.78)
Education			
≥ high school	**3.42(1.18; 5.66)**	-0.31(-1.75;1.14)	1.19(0.59;2.42)
Sex			
male	-0.11(-2.27; 2.05)	**-1.56(-2.97; -0.14)**	0.53(2.27;1.03)
Income			
500$—1499$	**3.25(0.62; 5.88)**	-0.88(-2.59;0.82)	0.81(0.36;1.80)
≥ 1500$	0.81(-3.09;4.71)	1.29(-1.3;3.87)	2.93(0.91;9.46)
Not reported	**3.72(1.17; 6.27)**	-0.50(-2.15;1.15)	0.80(0.38;1.72)
Marital Status			
married	0.02(-2.14;2.19)	-1.06(-1.51;1.30)	0.89(0.47;1.70)
Health Insurance			
Yes	-1.54(-3.51;0.43)	-0.01(-1.30;1.28)	1.23(0.66;2.28)
Employment Status			
employed	-0.71(-2.94;1.56)	-0.43(-1.91;1.05)	0.55(0.27;1.14)
Confident Filling out forms			
Yes	-1.37(-3.72;0.98)	0.21(-1.30;1.73)	0.91(0.45;1.83)
Material needs security	**-0.63(-1.13; -0.12)**	**-0.46(-0.78; -0.13)**	**0.82(0.69; 0.95)**

^*^ Linear Regression, Coefficients of associations (95% CI) are presented

^**^ Logistic Regression, Odds ratio (95% CI) are presented

Values in bold indicate statistical significance (*p* < 0.001)

Models adjusted for material needs security, age, sex, education, income, marital status, insurance, employment status, and Confident Filling out forms

## Discussion

To our knowledge, this is the first study that assessed the independent relationship between material needs insecurity and mental health outcomes, i.e., diabetes fatalism and depression, in a convenience sample of Lebanese adults with T2DM. Despite the current socioeconomic turmoil, our population possessed relatively high sum of material needs including a home, access to electricity, drinking water, television and cable subscription, internet access, computer, air condition and heating, telephone, and a car as a means for transportation. After adjusting for relevant confounding factors, the relationship between higher material needs security and mental health outcomes remained significant inferring that material needs security has a pivotal role on mental health outcomes. In addition, the study showed that middle age, having higher than a high school education and earning minimal income were strong and independent correlates of diabetes fatalism. However, being male was the only independent predictor of depression.

Understanding the underlying pathways of material needs subsets of SDOH and its association with adverse mental health highlights the dire need to recognize the social risk factors that are documented to impact adversely T2DM outcomes [2], particularly in underserved populations [2, 9, 11]. A systematic review proposed a conceptual framework that addresses material needs interaction with poor diabetes outcomes; insufficient income may lead to material needs insecurities which would induce adverse psychological health, such as depression [20], eventually compromising diabetes outcomes [6]. In our population, participants with lower sum of material needs were strongly associated with having adverse mental health outcomes (i.e. depression and diabetes fatalism). In a comparable underprivileged Lebanese population with T2DM, higher material needs security and being employed predicted better glycemic control; conversely, males were associated with poorer A1C [21, 22] which is comparable to Latino underprivileged population [23]. Despite the scarce evidence, studies in Lebanon suggest that possessing some material needs was significantly and independently associated with poor clinical outcomes among adults with T2DM in Lebanon.

In fact, socioeconomic status, comprising of income, education and occupation, has been the primary influencers and predictors of health outcomes at the individual and population level [20]. Accumulated research reveals that T2DM is socially graded whereby individuals with lower income and education are more prone to develop T2DM [4–8]. Material needs insecurity coupled with incapacity to make ends meet, chronic stress can surface leading to detrimental psychological responses such as depression. However, in our findings, socioeconomic status (income and education) did not predict depression, but rather fatalistic attitudes as measured by diabetes fatalism.

Fatalism, on the other hand, has been observed in underserved populations such as African Americans [17], Latinos [24] and even Arabs [14, 25]. As much as spirituality may have positive influences on health, there exists a divine belief that a higher power may intervene to manage diabetes and health generally [12]. Indeed, diabetes fatalism is linked to inadequate medication adherence, poor diet and lifestyle behaviors, uncontrolled glycemic levels, and decreased quality of life [5]. Our study revealed that middle age, having higher than a high school education and earning minimal income were strong and independent correlates of diabetes fatalism. Surprisingly, prior findings revealed that Lebanese adults with T2DM who exhibited more fatalistic attitudes were younger, and of lower education levels [13]. A possible explanation to these contradictory findings could be referred to the sociopolitical turmoil that Lebanon is currently undergoing. Adversity possibly strengthens people’s reliance on higher power as means of coping [12]. Evidently, the role of spirituality as a means of coping is perplexing; hence, investigating these factors as potential contributors particularly in dire circumstances is essential. The study findings provided baseline data on the existing relationship between SDOH subset, material needs, and mental health outcomes (diabetes fatalism and depression) for future focused studies on examining pathways and interrelationships between different SDOH drivers and diabetes outcomes. In addition, our preliminary data will inform our future directions to perform longitudinal assessments within Lebanon and expand to samples within the MENA region on the influences of social determinants and environmental risk factors in light of scarcity of data that are associated with diabetes health outcomes.

### Limitations

Inherent limitation of the convenience sampling of the study restricts broad generalizability of the findings; hence interpretation of study results need to be done with reservation and is limited to the context of our sample characteristics or any other similar population. Another inherent limitation is the cross-sectional design that limits inferring causality of relationships. The self-reporting nature of the questionnaires may have led to biases related to subjective interpretation of the questions, as well as recall and social desirability biases. Efforts to mitigate study limitations included using validated questionnaires to ensure reliable measurement, pilot testing to identify and correct ambiguities, and providing thorough training to researchers to reduce interviewer bias and ensure that participants understood the questions as intended. Participants were assured of anonymity and confidentiality to minimize social desirability bias. Additionally, regression analysis was employed to control for potential confounding variables, helping to isolate relationships between the primary variables of interest.

## Conclusion

Despite the emerging evidence on exploring SDOH in Western countries on the relationship between material needs [6–8] and mental health outcomes [11–14], there is scarce data that explores relationships between subsets of SDOH in the MENA region. With insufficient understanding of how SDOH interacts with diabetes outcomes, health care practitioners are not well equipped in tailoring diabetes care plan that best suits their patients [26]. Furthermore, findings from the current study can inform intervention programs for immigrant populations or refugees in many Western countries, including the United States of America with T2DM from the MENA region.

## Data Availability

Data will be made available on reasonable request.
